# Long Working Hours and the Risk of Glucose Intolerance: A Cohort Study

**DOI:** 10.3390/ijerph191811831

**Published:** 2022-09-19

**Authors:** Yesung Lee, Eunhye Seo, Woncheol Lee

**Affiliations:** Department of Occupational and Environmental Medicine, Kangbuk Samsung Hospital, School of Medicine, Sungkyunkwan University, Seoul 03181, Korea

**Keywords:** glucose intolerance, diabetes mellitus, long working hours, overwork, longitudinal studies, cohort studies

## Abstract

Long working hours have negative effects on the health of workers. Several studies have reported the association between long working hours and both diabetes and prediabetes. Therefore, we aimed to examine the temporal relationship between long working hours and glucose intolerance. Our cohort study collected data from 25,803 healthy male participants at baseline. To evaluate the risk of incident glucose intolerance, we estimated the hazard ratios (HRs) and 95% confidence intervals (CIs) using the Cox proportional hazards regression analyses. During 77,605.0 person-years of follow-up, 6741 participants developed glucose intolerance. Multivariable-adjusted HRs (95% CI) for weekly working 41–52 and >52 h compared with working 35–40 h, were 1.28 (1.17–1.40) and 2.80 (2.54–3.09), respectively. In the dose-response analyses, long working hours had a nearly linear relationship with the development of glucose intolerance across most working hours per week. The association between long working hours and incident glucose intolerance was stronger in the younger-age subgroups than in the older-age subgroups (*p* for interaction <0.001). Our large-scale cohort study demonstrated that long working hours were associated with incident glucose intolerance, with a dose-response relationship.

## 1. Introduction

Long working hours are an important public concern as it can threaten the health and well-being of workers [1]. Long working hours have a harmful effect on the health of workers and also lead to economic inefficiency. The World Health Organization (WHO) and the International Labour Organization (ILO) reported that 488 million people were exposed to long working hours in 2016, which was the occupational risk factor that caused the highest number of deaths (745,194 attributable deaths in 2016) [2,3]. According to the Organization for Economic Cooperation and Development (OECD) data, the average annual working hours in South Korea were 1915 h in 2021, meaning that it is one of the countries with the longest working hours among the OECD countries (the average in all OECD countries, 1716 h; the lowest in Germany, 1349 h) [4]. Due to the impact of coronavirus disease 2019 (COVID-19) lockdowns, working hours were reduced by 8.8% worldwide in 2020 compared to 2019. On the other hand, South Korea decreased by only 3.7% and returned to the level of 2019 in 2021 [4,5]. Therefore, in South Korea, the negative health impacts of long working hours are important issues that need to be addressed for the welfare of workers. 

Previous studies have reported that long working hours are not only associated with productivity losses [6], but also with health issues such as coronary heart disease [2,7], stroke [2,7], hypertension [8], obesity [9], and depression [10]. While these negative effects are related to diabetes, one of the major leading causes of death [11], the association between long working hours and diabetes remains controversial and inconsistent. A meta-analysis by Kivimäki et al. demonstrated that long working hours were linked to diabetes in workers with a low socioeconomic status [12]. However, there was a methodological limitation in that it included a large proportion of gray literature. Other studies reported no association between diabetes and long working hours [13,14]. Some studies showed different results according to gender [15,16] or shift work schedule [17]. On the other hand, a cross-sectional study by Baek et al. reported that only male workers had a relationship between long working hours and prediabetes, one of the major risk factors for diabetes [18]. 

Given that the prevalence of both diabetes and prediabetes in South Korea has been gradually increasing (from 6.6% and 7.9% in 2010, respectively, to 7.8% and 8.4% in 2030, respectively,) and 600 million people worldwide will live with diabetes in 2025, it is important to identify modifiable risk factors for glucose intolerance (both prediabetes and diabetes) to ensure the well-being of workers [11,19,20]. Hence, our longitudinal study aimed to elucidate the direct relationship between long working hours and incident glucose intolerance through a large-scale cohort of healthy South Korean male workers. 

## 2. Materials and Methods

### 2.1. Study Population

The Kangbuk Samsung Cohort Study is a cohort study of South Korean adults aged at least 18 years, who underwent a comprehensive annual or biennial health examination at the Kangbuk Samsung Hospital Total Healthcare Center in Seoul and Suwon, South Korea [21]. Most of the participants were employees of companies in various industries and local governmental organizations and their spouses. In South Korea, the Industrial Safety and Health Law requires free-of-charge annual or biennial health screening examinations for all employees.

**Figure 1:**
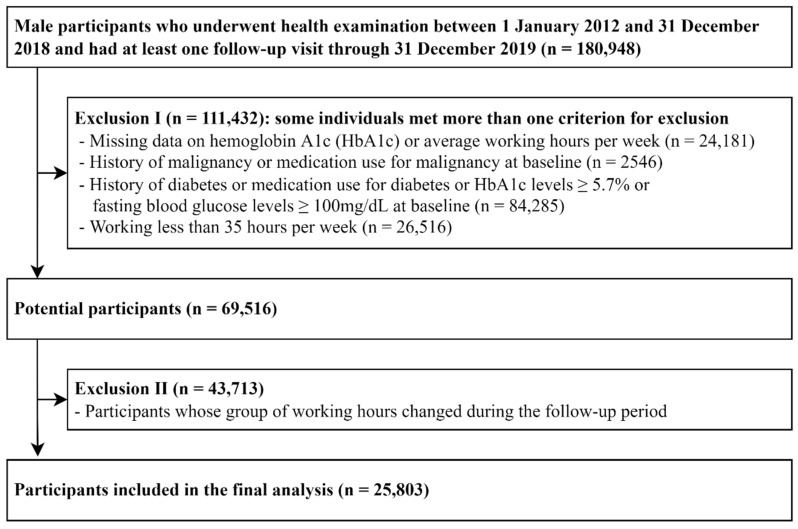
Flowchart of study participants.

The present study included a total of 180,948 male participants who underwent health examinations from 1 January 2012 to 31 December 2018 and had undergone at least one other screening examination before 31 December 2019. First, we excluded 111,432 participants who met any of the following exclusion criteria at baseline (Figure 1): missing data on hemoglobin A1c (HbA1c) or average working hours per week, history of malignancy or medication use for malignancy, history of diabetes or medication use for diabetes, HbA1c levels ≥ 5.7% or fasting blood glucose levels ≥ 100 mg/dL, and working less than 35 h per week. Second, among the potential participants of 69,516 non-diabetic male workers, we further excluded 43,713 workers whose group of working hours changed during the follow-up period because of the fluctuation in their weekly working hours. Finally, 25,803 participants were eligible for our study at baseline. 

This study was approved by the Institutional Review Board (IRB) of Kangbuk Samsung Hospital, and the requirement for informed consent was waived because we accessed only de-identified data routinely collected as part of the health screening examinations (IRB No: KBSMC2022-08-050). The data are not available to be shared publicly because we do not have permission from the Institutional Review Board to distribute the data. However, data from the Kangbuk Samsung Cohort Study can be made available upon reasonable request to the authors of the study and who may be contacted using the corresponding author contact details in this manuscript. 

### 2.2. Measurements

All examinations were conducted at the Kangbuk Samsung Hospital Total Healthcare Screening Center. At each visit, the demographic characteristics, smoking status, alcohol consumption, regular exercise, education level, monthly household income, marital status, medical history, and medication use were collected by using standardized, self-administered questionnaires [21]. Smoking status was categorized as not current or current smoker. Alcohol consumption for heavy drinking was categorized as ≥30 g/day for men and ≥20 g/day for women. We assessed the weekly frequencies of vigorous leisure-time physical activity. Regular exercise was defined as exercising three or more times per week. Education level was categorized as less than college or college graduate or higher. Monthly household income was categorized as <6 million Korean Republic won (KRW) per month or ≥6 million KRW per month. Marital status was categorized as married or not married. 

Working hours were assessed using the following question: “How many hours did you work in a week on average in your job for the past year, including overtime?” According to the Labor Standards Act of Korea, the working hours of adults should not exceed 40 h per week, except for recess time (12 additional working hours per week are permitted with employee consent). Based on the International Labour Office report, individuals who worked less than 35 h per week were defined as part-time workers [1]. We excluded part-time workers to minimize deviations in our evaluation of the health effects of long working hours. In this regard, the average weekly working hours over the past year were categorized as 35–40, 41–52, and >52 h per week. The shift work schedule was assessed using the following question: “In the past year, during which time of the day did you work the most?” Daytime work was defined as work performed mostly during the day (between 6 AM and 6 PM), while shift work was defined as work performed during other hours. 

Blood pressure, weight, and height were measured by trained nurses. Obesity was defined as body mass index (BMI) ≥25 kg/m^2^. The fasting blood sample measurements included glucose, total cholesterol, low-density lipoprotein cholesterol (LDL-C), high-density lipoprotein cholesterol (HDL-C), triglycerides, and high-sensitivity C-reactive protein (hsCRP). Insulin resistance was assessed using the homeostasis model assessment of insulin resistance (HOMA-IR) equation as follows: fasting insulin (μU/mL) × fasting glucose (mg/dL)/405 [22]. 

HbA1c levels were measured using an immunoturbidimetric assay with a Cobra Integra 800 automatic analyzer (Roche Diagnostics, Basel, Switzerland). In general, fasting blood glucose and HbA1c are equally appropriate for diagnostic screening. Nevertheless, HbA1c has several advantages, including greater convenience (fasting not required), greater preanalytical stability, and less day-to-day variability [23]. In accordance with the American Diabetes Association standards, prediabetes was defined as HbA1c level between 5.7% and 6.4%, and diabetes was defined as HbA1c level ≥6.5% [23]. For our longitudinal study, we defined incident glucose intolerance as HbA1c level ≥5.7%, including the development of diabetes during the follow-up period.

### 2.3. Statistical Analysis

The baseline characteristics of study participants were presented according to the three groups of weekly working hours. Descriptive statistics were used to summarize the characteristics of participants categorized by the groups of working hours. The primary endpoint was the development of incident glucose intolerance. Participants were followed up from baseline to the endpoint visit, or to the last available visit until 31 December 2019, whichever came first. Incidence density was calculated as the number of incident cases divided by person-years of follow-up. 

The hazard ratios (HRs) and 95% confidence intervals (CIs) for incident glucose intolerance were estimated by using Cox proportional hazards regression analyses. Initially, only age was adjusted in the crude model. Model 1 was adjusted for age, alcohol intake, smoking status, regular exercise, education level, marital status, and household income. To adjust for potential confounders, Model 2 was further adjusted for medication for hypertension, dyslipidemia, BMI, HOMA-IR, and hsCRP. Lastly, to adjust for other occupational risk factors, Model 3 was further adjusted for shift work schedules. The proportional hazards assumption was assessed by examining graphs of estimated log (-log) survival and by using the ‘*estat phtest*’ command based on Schoenfeld residuals; no violation of the assumption was found. To demonstrate the linear trend of incidence, the number of groups was used as a continuous variable and examined in each model. 

To further explore the dose-response relationship between long working hours and the risk of glucose intolerance, we conducted two dose-response analyses. First, HRs were estimated with 95% CIs associated with a 1-h increase in weekly working hours and were used as a continuous variable in the regression models. Second, restricted cubic splines with knots were performed at the 5th, 27.5th, 50th, 72.5th, and 95th percentiles of baseline weekly working hours distribution. To explore whether the associations between long working hours and glucose intolerance differed according to risk factors for diabetes, subgroup analyses were performed according to age (<40 versus ≥40 years), BMI (<25 versus ≥25 kg/m^2^), and HOMA-IR (<2.5 versus ≥2.5). Interactions between the groups of working hours and subgroup characteristics were tested using likelihood ratio tests, which compared models with and without multiplicative interaction terms. 

In sensitivity analysis, to test the robustness of our primary outcomes, we included 43,713 participants whose group of working hours changed during the follow-up period. For the 69,516 workers, Cox proportional hazards regression analyses were performed in the same manner as in the main analysis. 

Statistical analyses were performed using STATA version 17.0 (StataCorp LP, College Station, TX, USA). All reported *p* values were two-tailed and *p* values <0.05 were considered statistically significant. 

## 3. Results

As shown in Table 1, the mean (standard deviation) age, fasting blood glucose level, and HbA1c level at baseline were 36.6 (7.6) years, 91.1 (5.5) mg/dL, and 5.39 (0.19) %, respectively. Weekly working hours were positively associated with BMI and HOMA-IR. In contrast, weekly working hours were negatively associated with regular exercise, systolic blood pressure, and daytime work. 

**Figure 2:**
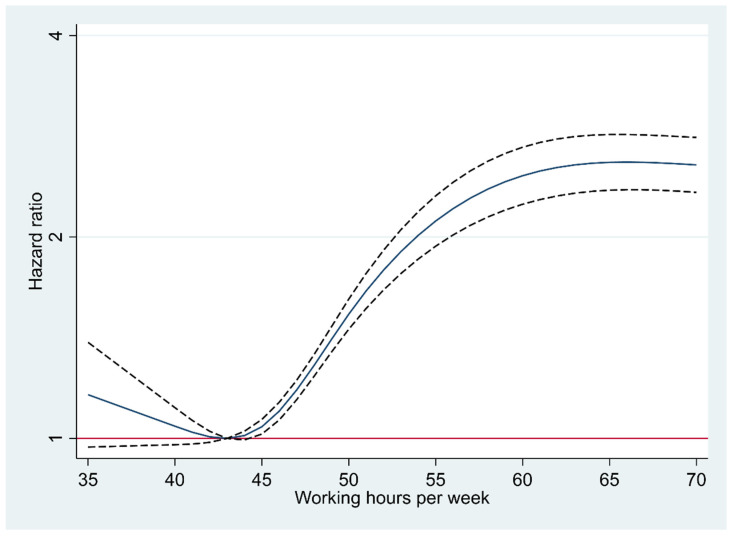
Multivariable-adjusted hazard ratio for glucose intolerance with the restricted cubic spline (70 h; 95th percentile of weekly working hours).

Table 2 shows the association between long working hours and the risk of incident glucose intolerance. Among a total of 25,803 participants, there were 6741 incident cases of glucose intolerance (incidence density, 8.69 per 100 person-years) over 77,605.0 person-years of follow-up (mean follow-up, 3.0 years). All models indicated a significantly higher risk of glucose intolerance in all groups of longer working hours compared with the reference group (working 35–40 h per week). In Model 3, introducing all potential confounders considered in the study, multivariable-adjusted HRs (95% CI) of incident glucose intolerance for weekly working 41–52 h and >52 h, compared with working 35–40 h, were 1.28 (1.17–1.40) and 2.80 (2.54–3.09), respectively. When working hours were treated as a continuous variable in regression models, the HR (95% CI) associated with a one hour increase in Model 3 was 1.03 (1.02–1.03). Moreover, in a multivariable-adjusted spline regression model, there was a significant dose-response relationship between weekly working hours and the development of glucose intolerance across most ranges of working hours (Figure 2).

In subgroup analyses (Table 3), the association between weekly working more than 52 h and incident glucose intolerance, compared with working 35–40 h, remained consistent and significant among participants in all clinically relevant subgroups. Meanwhile, the association was significantly stronger among participants aged < 40 years than among those aged ≥40 years (*p* for interaction < 0.001). 

In sensitivity analysis (Table S1), when Cox proportional hazards regression analyses were performed for 69,516 participants, including workers whose group of working hours had changed during the follow-up period, the association between weekly working more than 52 h at baseline and incident glucose intolerance, compared with working 35–40 h, was still observed with borderline significance in Model 3. 

## 4. Discussion

In this large-scale cohort study, our results demonstrated that non-diabetic participants with long working hours had a high risk of developing glucose intolerance in a dose-response manner. In particular, consistently working more than 52 h per week was associated with a 2.8-fold higher risk of incident glucose intolerance compared with working 35–40 h per week. Furthermore, after stratification into predetermined subgroups, the association remained significant in all subgroups. In sensitivity analysis, including workers whose weekly working hours changed during follow-up, the association was slightly attenuated but still borderline significant. 

To our knowledge, the mechanism underlying the relationship between long working hours and glucose intolerance remains unknown. However, it is reasonable to assume that the mechanism is similar to that between long working hours and diabetes, especially type 2 diabetes. Several mechanisms can be broadly classified into two categories: behavioral and biological mechanisms. Regarding behavioral risk factors, long working hours are associated with increased negative health-related behaviors, such as alcohol consumption, smoking, physical inactivity, and sleep deprivation [24,25]. It is well-known that smoking, as a predictor of the progression of glucose intolerance in a dose-dependent manner, is a major risk factor for diabetes [26,27]. Binge and heavy drinking are also risk factors for diabetes [28]. In addition, long working hours are related to prolonged sedentary time [29], resulting in decreased leisure-time physical activity [7], which interact with obesity to increase the risk of diabetes [30]. On the other hand, in our study, there was no interaction between long working hours and obesity for the development of glucose intolerance. With regard to sleep, insufficient sleep duration is associated with an increased risk of diabetes [31]. Specifically, insufficient sleep duration causes insulin resistance, reduced β-cell activity, and an increased caloric intake because of dysregulations in the neuroendocrine system, such as decreased leptin, increased ghrelin, and decreased sympathetic activity. Furthermore, a recent cross-sectional study by Min et al., reported that long working hours were linked to an insufficient intake of dietary fiber [32]. Dietary fiber intake improves insulin resistance and alleviates obesity, thereby reducing the risk of diabetes [33]. However, our results showed that long working hours did not interact with obesity and insulin resistance in glucose intolerance, suggesting that fiber diet may not play a major role in modifying the association between long working hours and glucose intolerance. 

With respect to biological mechanisms, chronic stress response caused by long working hours could result in glucose intolerance. Long working hours can cause work-related stress, such as job strain [34]. Physiological responses to such psychological stress are related to new-onset type 2 diabetes [35]. Sustained stress exposure leads to chronic activation of the hypothalamic–pituitary–adrenal axis which in turn stimulates the release of cortisol. Dysregulated cortisol output affects insulin sensitivity by decreasing insulin secretion through glucocorticoid receptors expressed by pancreatic β-cells [36]. Eventually, such neuroendocrine dysfunction increases the susceptibility to hypoglycemia and the risk of diabetes [35,37]. In a cohort study by Hackett et al., a flat slope of cortisol levels across the day and elevated evening cortisol levels were predictive of impaired fasting glucose, including incident diabetes, similar to our primary outcome [38]. Additionally, stress activates the sympathetic nervous system, which increases blood pressure affecting insulin sensitivity [35,39]. Lastly, stress triggers chronic low-grade inflammation by proinflammatory cytokines, in turn influencing the risk of developing diabetes [35,40]. 

In our subgroup analyses, the association between more than weekly working 52 h and incident glucose intolerance was stronger in the younger-age subgroups than in the older-age subgroups. Age is a well-known risk factor for diabetes, and the prevalence of glucose intolerance increases with age [19]. Despite the physiological changes that make people more susceptible to diabetes with aging [41], our results suggested that younger individuals with long working hours were more vulnerable to glucose intolerance. Several reasons can be attributed to the contrast between our results and the general expectations. First, the risk of young-onset (diagnosis at age < 40 years) type 2 diabetes increases as the number of metabolic syndrome components increases [42]. Epidemiological studies have reported an association between long working hours and metabolic syndrome, but this association remains controversial [43]. Although various metabolic abnormalities were adjusted in our study, the association was still significant, suggesting that that other pathways may be involved. Second, A 2021 report from the Bureau of Labor Statistics, a division of the U.S. Department of Labor, showed that the younger the workers, the lower their wages. Furthermore, according to the European Agency for Safety and Health at Work, young workers are more likely to suffer a work-related injury than older workers. In addition to these stressful situations, young workers tend to experience significant work-related stress due to their characteristics, such as lack of life experience, lack of other options, impatience, and skepticism [44]. Therefore, since young workers are already prone to stress, the risk of glucose intolerance would likely increase if the situation of long working hours was added. Third, the older the participants, the more likely they were to be initially excluded because they were undergoing treatment or met the diagnostic criteria of diabetes, so the healthy worker effect cannot be ruled out from the older subgroup. Therefore, further studies are required to elucidate the underlying mechanisms. 

There are several limitations that need to be considered in this study. First, weekly working hours and covariates were collected by self-administered questionnaires; therefore, observational errors could not be excluded. However, overreporting or underreporting had no benefit to the examinees. Hence, the results were not affected by differential misclassification. Second, the study population was limited to males. However, women had to be excluded because the data violated the proportional hazards assumption, which would lead to a high probability of unnecessary misinterpretation. Therefore, our findings can only be applied to men. Third, the participants were young and middle-aged Koreans with relatively good health and a high educational level. Accordingly, our results may not be generalized to other populations by age, race, or ethnicity. Lastly, other occupational variables, such as occupational stress, occupational physical activity, occupational sedentary time, type of collar work, type of shift work, or other life-style variables, such as diet and sleep quality, were not evaluated in this study. Previous studies have shown that the prevalence and incidence of diabetes in blue-collar workers and unskilled workers are higher than in white-collar workers and professionals [45,46,47]. The underlying explanations could be nutritional status due to differences in socioeconomic status, physical and psychological stress due to stressful work environments, lack of knowledge due to education level, unhealthy behaviors such as smoking and drinking, and poor access to medical care. If these unmeasured variables are included in further studies, the evidence obtained will help clarify the underlying mechanisms. Meanwhile, the extent to which a potential unmeasured confounder negated the observed association between long working hours and glucose intolerance can be assessed by calculating the E-value [48]. The E-value indicates the robustness of our main findings to unmeasured confounding. In Model 3, the measured E-value for weekly working more than 52 h was 5.04 (95% CI = 4.52–5.63), suggesting that the observed HR of 2.80 could be explained away by an unmeasured confounder that was associated with both our exposure and outcome by a HR of 5.04-fold each, above and beyond the measured confounders, but weaker confounding could not do so; an unmeasured confounder that was associated with both our exposure and the outcome by a HR of 4.52-fold each, above and beyond the measured confounders, could shift the CI to include the null, but weaker confounding could not do so [48,49]. 

Despite these limitations, this study has notable strengths. First, to our knowledge, this is the first longitudinal study with a large sample size to evaluate the temporal association between long working hours and the risk of incident glucose intolerance in a dose-response manner. Second, this study definitively and strictly assessed the negative health effect of sustained long working hours by evaluating the fixed exposure of working hours. If the analyses were based on working hours at baseline as in previous studies, the results would have been underestimated, as shown in Table S1. Finally, our cohort consisted of relatively young and middle-aged healthy men, suggesting a low susceptibility to survivor bias owing to comorbidities. 

## 5. Conclusions

Our large-scale cohort study demonstrated that long working hours were associated with the development of glucose intolerance in a dose-response relationship. It is crucial to find preventable negative effects of long working hours on the health of workers. 

## Figures and Tables

**Table 1:** Baseline Characteristics of study participants by weekly working hours.

		Weekly Working Hours			*p* for Trend
Characteristics	Overall	35–40	41–52	>52	
Number	25,803	5171	16,316	4316	
Age (years) *	36.6 (7.6)	40.4 (9.3)	35.3 (6.6)	36.7 (7.1)	<0.001
Current smoker (%)	30.0	33.0	26.8	38.2	0.002
Heavy Alcohol intake (%) ^a^	18.8	23.8	16.5	21.3	<0.001
Regular exercise (%) ^b^	14.8	18.0	14.6	11.5	<0.001
High education level (%) ^c^	89.9	83.2	92.5	88.0	<0.001
Marital status—married (%)	69.6	78.7	66.0	72.0	<0.001
High household income (%) ^d^	30.4	32.6	28.2	35.8	0.223
Medication for hypertension (%)	3.27	5.67	2.45	3.54	<0.001
Medication for dyslipidemia (%)	1.76	2.61	1.40	2.13	0.029
Obesity (%) ^e^	34.1	34.2	33.3	36.9	0.011
BMI (kg/m^2^) *	24.1 (2.8)	24.1 (2.7)	24.1 (2.8)	24.4 (2.9)	<0.001
Systolic BP (mmHg) *	112.5 (10.7)	113.0 (11.1)	112.4 (10.6)	112.0 (10.6)	<0.001
Diastolic BP (mmHg) *	72.2 (8.8)	73.3 (9.1)	71.8 (8.7)	72.2 (8.9)	<0.001
Glucose (mg/dL) *	91.1 (5.5)	91.5 (5.4)	90.9 (5.5)	91.1 (5.6)	<0.001
Hemoglobin A1c (%) *	5.39 (0.19)	5.39 (0.19)	5.38 (0.19)	5.41 (0.18)	<0.001
HOMA-IR ^#^	1.22 (0.82–1.76)	1.19 (0.80–1.72)	1.22 (0.83–1.77)	1.23 (0.83–1.78)	0.021
Total cholesterol (mg/dL) *	194.5 (32.8)	196.0 (33.7)	193.6 (32.5)	195.8 (32.8)	0.498
LDL-C (mg/dL) *	126.2 (30.6)	127.7 (30.9)	125.6 (30.5)	126.6 (30.5)	0.032
HDL-C (mg/dL) *	54.6 (13.3)	54.5 (13.4)	54.9 (13.3)	54.0 (13.2)	0.161
Triglycerides (mg/dL) ^#^	100 (72–144)	103 (74–149)	99 (71–142)	101 (74–145)	0.102
hsCRP (mg/L) ^#^	0.05 (0.03–0.09)	0.05 (0.03–0.09)	0.05 (0.03–0.09)	0.05 (0.03–0.09)	0.06
Daytime work (%) ^f^	89.8	90.8	90.6	85.5	<0.001

Data are expressed as * mean (standard deviation), ^#^ median (interquartile range), or percentage. ^a^ ≥30 g/day; ^b^ ≥3 times/week; ^c^ ≥College graduate; ^d^ ≥6 million KRW per month; ^e^ BMI ≥ 25 kg/m^2^; ^f^ Participants who answered “I work mostly during the day (between 6 AM and 6 PM)”; BMI, body mass index; BP, blood pressure; HOMA-IR, homeostasis model assessment of insulin resistance; LDL-C, low-density lipoprotein cholesterol; HDL-C, high-density lipoprotein cholesterol; hsCRP, high-sensitivity C-reactive protein; KRW, Korean Republic won.

**Table 2:** Incidence and risk of glucose intolerance according to weekly working hours.

Weekly Working Hours	Person-Years (PY)	Incident Cases	Incidence Density (per 100 PY) (95% CI)	Age-Adjusted HR (95% CI)	Multivariable-Adjusted HR (95% CI) ^a^		
					Model 1 *	Model 2 **	Model 3 ***
35–40	15,646.9	1194	7.63 (7.21–8.08)	1.00 (reference)	1.00 (reference)	1.00 (reference)	1.00 (reference)
41–52	51,421.0	3858	7.50 (7.27–7.74)	1.32 (1.23–1.41)	1.33 (1.23–1.44)	1.29 (1.18–1.41)	1.28 (1.17–1.40)
>52	10,537.1	1689	16.03 (15.28–16.81)	2.65 (2.46–2.86)	2.72 (2.50–2.97)	2.79 (2.53–3.08)	2.80 (2.54–3.09)
per 1 h				1.02 (1.02–1.03)	1.02 (1.02–1.03)	1.03 (1.02–1.03)	1.03 (1.02–1.03)
*p* for trend				<0.001	<0.001	<0.001	<0.001

^a^ Estimated from Cox proportional hazard models; * Model 1 was adjusted for age, alcohol intake, smoking status, regular exercise, education level, marital status, and household income; ** Model 2: model 1 plus adjustment for medication for hypertension, medication for dyslipidemia, BMI, HOMA-IR, and hsCRP; *** Model 3: model 2 plus adjustment for shift work schedule; HR, hazard ratio; CI, confidence interval; BMI, body mass index; HOMA-IR, homeostasis model assessment of insulin resistance; hsCRP, high-sensitivity C-reactive protein.

**Table 3:** Hazard ratios ^a^ (95% CI) for glucose intolerance by weekly working hours in clinically relevant subgroups.

Subgroup	Weekly Working Hours			*p* for Trend	*p* for Interaction
	35–40	41–52	>52		
Age					<0.001
<40 years (n = 18,041)	1.00 (reference)	1.10 (0.97–1.24)	2.72 (2.38–3.10)	<0.001	
≥40 years (n = 7762)	1.00 (reference)	1.32 (1.16–1.50)	2.27 (1.96–2.63)	<0.001	
BMI					0.317
<25 kg/m^2^ (n = 16,996)	1.00 (reference)	1.28 (1.14–1.44)	2.91 (2.56–3.31)	<0.001	
≥25 kg/m^2^ (n = 8800)	1.00 (reference)	1.28 (1.12–1.48)	2.67 (2.28–3.11)	<0.001	
HOMA-IR					0.557
<2.5 (n = 23,419)	1.00 (reference)	1.29 (1.18–1.42)	2.80 (2.52–3.11)	<0.001	
≥2.5 (n = 2310)	1.00 (reference)	1.20 (0.92–1.56)	2.84 (2.13–3.79)	<0.001	

^a^ Estimated from Cox proportional hazard models adjusted for age, alcohol intake, smoking status, regular exercise, education level, marital status, household income, medication for hypertension, medication for dyslipidemia, BMI, HOMA-IR, hsCRP, and shift work schedule; CI, confidence interval; BMI, body mass index; HOMA-IR, homeostasis model assessment of insulin resistance; hsCRP, high-sensitivity C-reactive protein.

## Data Availability

The data are not available to be shared publicly, because we do not have permission from the Institutional Review Board to distribute the data. However, data can be made available from the Kangbuk Samsung Cohort Study upon reasonable request, by contacting the corresponding authors of this manuscript.

## Supplementary Materials

The following supporting information can be downloaded at: https://www.mdpi.com/article/10.3390/ijerph191811831/s1, Table S1: Development of glucose intolerance according to weekly working hours including participants who changed group of working hours during follow-up.
