# Supplementary material for: Long Working Hours and the Risk of Glucose Intolerance: A Cohort Study
Source: Int J Environ Res Public Health. 2022 Sep 19;19(18):11831. doi: 10.3390/ijerph191811831 (PMC9517219; doi:10.3390/ijerph191811831)
Supplement: Supplementary file 1 [file ijerph-19-11831-s001.zip › ijerph-1908625-supplementary.pdf]

Table S1. Incidence and risk of glucose intolerance according to weekly working hours including participants who changed group of working hours during follow-up

Weekly working hours	Person-years (PY)	Incident cases	Incidence density (per 100 PY) (95% CI)	Age-adjusted HR (95% CI)	Multivariable-adjusted HR (95% CI) [a]		
					Model 1*	Model 2**	Model 3***
35-40	38,491.8	2,075	5.39 (5.16-5.63)	1.00 (reference)	1.00 (reference)	1.00 (reference)	1.00 (reference)
41-52	150,289.8	7,510	5.00 (4.89-5.11)	1.07 (1.02-1.13)	1.06 (1.00-1.12)	1.06 (0.99-1.13)	1.05 (0.99-1.12)
>52	84,120.1	4,306	5.12 (4.97-5.27)	1.11 (1.06-1.18)	1.10 (1.04-1.17)	1.08 (1.01-1.15)	1.07 (1.00-1.14)
per 1 hour				1.003 (1.001-1.004)	1.003 (1.001-1.01)	1.00 (1.00-1.004)	1.00 (1.00-1.004)
P for trend				<0.001	0.002	0.052	0.088

[a] Estimated from Cox proportional hazard models. * Model 1 was adjusted for age, alcohol intake, smoking status, regular exercise, education level, marital status, and household income. ** Model 2: model 1 plus adjustment for medication for hypertension, medication for dyslipidemia, BMI, HOMA-IR, and hsCRP. *** Model 3: model 2 plus adjustment for shift work schedule. HR, hazard ratio; CI, confidence interval; BMI, body mass index; HOMA-IR, homeostasis model assessment of insulin resistance; hsCRP, high-sensitivity C-reactive protein
